# Efficacy and safety of bright light therapy for manic and depressive symptoms in patients with bipolar disorder: A systematic review and meta‐analysis

**DOI:** 10.1111/pcn.12976

**Published:** 2020-02-10

**Authors:** Masahiro Takeshima, Tomohiro Utsumi, Yumi Aoki, Zhe Wang, Masahiro Suzuki, Isa Okajima, Norio Watanabe, Koichiro Watanabe, Yoshikazu Takaesu

**Affiliations:** ^1^ Department of Neuropsychiatry Akita University Graduate School of Medicine Akita Japan; ^2^ Department of Psychiatry Jikei University School of Medicine Tokyo Japan; ^3^ Psychiatric & Mental Health Nursing St. Luke's International University Tokyo Japan; ^4^ Renaissance School of Medicine at Stony Brook University New York USA; ^5^ Department of Psychiatry Nihon University School of Medicine Tokyo Japan; ^6^ Department of Psychological Counseling, Faculty of Humanities Tokyo Kasei University Tokyo Japan; ^7^ Department of Health Promotion and Human Behavior and of Clinical Epidemiology Kyoto University Graduate School of Medicine Kyoto Japan; ^8^ Department of Neuropsychiatry Kyorin University School of Medicine Tokyo Japan

**Keywords:** bipolar disorder, bright light therapy, chronotherapy, depression, phototherapy

## Abstract

**Aim:**

This systematic review and meta‐analysis evaluated whether bright light therapy (BLT) is an effective and safe treatment for manic/depressive symptoms and a preventive measure for recurrent mood episodes in patients with bipolar disorder.

**Methods:**

A literature search of major electronic databases was conducted in June 2019, including all published articles up to that date. Two researchers independently selected relevant publications, extracted data, and evaluated methodological quality according to the Cochrane criteria.

**Results:**

Six randomized controlled trials (RCT) evaluated the efficacy of BLT for bipolar depression. A meta‐analysis found no significant differences between BLT and placebo for the following outcomes: (i) rates of remission from depressive episodes (risk ratio [RR]: 1.81, 95% confidence interval [CI]: 0.43 to 7.64, *P* = 0.42); (ii) depressive symptom scores (standardized mean difference: −0.25, 95%CI: −0.74 to 0.23, *P* = 0.30); and (iii) rates of manic switching (RR: 1.00, 95%CI: 0.28 to 3.59, *P* = 0.26). The sensitivity analysis for studies with low overall indirectness did show a significant antidepressant effect for BLT (RR: 3.09, 95%CI: 1.62 to 5.90, *P* = 0.006). No RCT investigated the effect of BLT in preventing the recurrence of mood episodes in the euthymic state or in improving manic symptoms in the manic state. No severe adverse events were reported.

**Conclusion:**

While a meta‐analysis was unable to demonstrate the efficacy of BLT for bipolar depression, a sensitivity analysis did show a significant effect. Further well‐designed studies are needed to clarify the effectiveness of BLT, not only for the depressive state but also for other states, in the treatment of bipolar disorder.

Bipolar disorder (BD) is characterized by alternating episodes of depression and mania or hypomania.^1^, ^2^ Patients often experience recurrent mood symptoms despite receiving pharmacological treatment.^3^ Psychosocial interventions, such as psychoeducation, cognitive behavioral therapy, interpersonal and social rhythm therapy, and family therapy, can improve mood symptoms and prevent relapse.^4^ However, these treatments require skilled therapists, and the evidence for the efficacy of psychosocial therapy for BD is inconsistent.^5^ As BD treatment requires a multidisciplinary approach, novel therapeutics are continuously being explored.^5^, ^6^

Circadian rhythm dysfunction is one of the most common symptoms in patients with BD.^7^ A recent review suggested that circadian rhythm dysfunction is more prominent in patients with BD than in those with major depression.^8^ In those with BD, the need for sleep generally decreases during the manic phase and increases during the depressive phase. A delayed sleep–wake phase has also been reported in bipolar depression.^9^ Moreover, studies show that circadian rhythm dysfunction can be a predictor for shorter times to relapse,^10^ prolonged mood episodes,^11^ and more mood and motor variability during euthymic periods.^12^ Thus, interventions that focus on improving circadian rhythm dysfunction could lead to positive outcomes on depressive symptoms and the prevention of relapse of mood episodes.

Chronotherapy has been developed over the past 50 years, and comprises bright light therapy (BLT), sleep deprivation (SD), and sleep phase advance therapy.^13^ Among these chronotherapies, BLT has been shown to be the most versatile and is widely used to treat depressive symptoms in seasonal affective disorders^14^ and nonseasonal major depression.^15^ Although the exact mechanism is unknown, bright light is believed to modulate circadian rhythm dysfunction and autonomic functions and consequently improve mood symptoms.^16^ In recent years, an increasing number of randomized controlled trials (RCT) have investigated the effectiveness of BLT for depressive symptoms in patients with BD. Some RCT have suggested that BLT is effective,^17^, ^18^ while others have shown no therapeutic effect. The International Society of Bipolar Disorders (ISBD) Task Force on Chronobiology and Chronotherapy recommended BLT for the acute phase of bipolar depression, and concluded that BLT had the strongest evidence among current chronotherapeutic options.^19^ However, only one meta‐analysis has examined the effects of BLT on bipolar depression.^20^ Tseng *et al*. reported that treatment with BLT had statistically significant antidepressant effects and did not increase mood polarity compared to treatment without BLT.^21^ However, this meta‐analysis had some limitations that might have affected its results: (i) most included studies were not RCT; (ii) articles were limited to the English language; (iii) databases other than PubMed and http://clinicaltrials.gov were not searched; and (iv) one study consisted of 70% of subjects with major depressive disorder.^22^ In addition, recent RCT that examined the efficacy of BLT for BD were not included in this meta‐analysis.^17^, ^18^ Importantly, no meta‐analysis so far has examined the efficacy of BLT for manic states, or for preventing the recurrence of mood episodes in BD.

In this systematic review and meta‐analysis of RCT, we therefore aimed to evaluate the efficacy of BLT for manic/depressive symptoms and for preventing recurrent mood episodes in patients with BD. We also evaluated adverse events, especially manic switching, associated with BLT in BD.

## Methods

This study was conducted in accordance with the PRISMA recommendations for reporting systematic reviews and meta‐analyses,^23^ and preregistered with PROSPERO (registration number: CRD 42019141232).^24^

### Search strategy

We searched the electronic databases Ovid MEDLINE (search date: 24 June 2019), Cochrane Central Register of Controlled Trials (CENTRAL; 24 June 2019), Embase (24 June 2019), PsychINFO (25 June 2019), and http://clinicaltrials.gov (4 July 2019) for reports of RCT using appropriate subject headings and relevant search terms (e.g., ‘bipolar disorder,’ ‘phototherapy,’ ‘randomized controlled trial’; see Table S1). When necessary, we contacted the authors of specific studies to clarify additional points.

### Inclusion criteria

Studies in any language that met the following criteria were included in the final review:

1. RCT at the individual or cluster level. Crossover studies were included if they reported results during the first period (i.e., before the crossover) as a carry‐over effect of treatment that might affect the subsequent periods.
2. Patients (in any mood state) with a clinical diagnosis of BD, type I or type II, according to the diagnostic criteria used in the specific study (diagnosed using any recognized diagnostic criteria).
3. At least 80% of the participants were diagnosed with BD, type I or type II.
4. Interventions comprised any kind of light therapy, such as ‘light therapy,’ ‘bright light therapy,’ ‘phototherapy,’ or chronotherapy in any intensity and color.
5. Control groups comprised sham treatment (such as low‐intensity light, dim red light, or negative ion) or treatment as usual (no light treatment).

### Article selection process

Author Y. A. removed duplicates prior to eligibility screening. Subsequently, two groups of screeners, with two authors in each group, were created (group 1: M. T. and T. U.; group 2: Y. A. and Z. W.). In each group, the two authors independently screened the titles and abstracts of the identified articles, and excluded studies on the basis of the above criteria. Reasons for exclusion were registered by the authors in each group. Any disagreement between the screeners was resolved by author Y. T. after thorough and systematic discussions. After identifying eligible studies, the full text of each study was examined.

### Outcome measures

The primary outcome measures included the following: (i) rates of remission from depressive or manic episodes; (ii) rates of relapse from euthymic states; and (iii) changes in scores on depression or mania rating scales. Remission from depressive episodes was defined as a score for the Structured Interview Guide for the Hamilton Depression Rating Scale with Atypical Depression Supplement (SIGH‐ADS) ≤ 8, for the 17‐item Hamilton Depression Rating Scale (HAM‐D_17_) ≤ 7, or for the 21‐item Hamilton Depression Rating Scale (HAM‐D_21_) ≤ 8, depending on the specific outcome measure used in a study. Remission from manic episodes was defined as a score ≤ 12 on the Young Mania Rating Scale. Rates of remission from depressive/manic episodes were calculated by dividing the number of participants who achieved remission in a group by the total number of participants in that group. When remission rates from depressive/manic symptoms were not reported in an article, we calculated the rate based on the reported depressive/manic symptoms (mean ± standard deviation) using Excel.

The secondary outcome measures included the following: (iv) improvements in daytime functioning; and (v) improvements in insomnia symptoms after the intervention. Other outcome measures included suicide rates, incidences and rates of manic switching, and any incidences of adverse events. However, definitions of manic switching were not consistent across studies.

When a three‐arm study included two different control groups, we selected only one control group with the following priority: (i) the group with sham treatment, such as dim light; and (ii) the group with the lower dropout rate.

### Study quality and risk‐of‐bias assessment

Six authors were divided into three groups (group 1: M. T. and M. S., group 2: I. O. and Y. A., group 3: T. U. and Z. W.) to evaluate the quality of the studies and assess their risk of bias. The authors in each group carefully and independently extracted the relevant data. Author Y. T. performed checks to ensure quality and consistency of the assessment. The following variables were extracted from each study: demographics of the participants (e.g., education, employment status, marital status, family members); diagnostic criteria for BD; details on the participants’ BD history (type, age of onset, family history, number of mood episodes); details on the BD treatment (e.g., mood stabilizers, antipsychotics, antidepressants); concurrent psychiatric disorders; country in which the study was performed; depressive and manic symptoms; and definitions of remission from depressive/manic episodes, manic switching from depressive or euthymic states, relapse from euthymic states, daytime functioning, and insomnia, if reported. The following additional variables were also recorded: RCT type; study settings (primary or secondary care); inclusion and exclusion criteria of participant recruitment; contents of the intervention (timing, duration, intensity, color); contents of the control group intervention; quality assurance of the intervention; funding source; and numbers of dropouts from the intervention and control groups. The quality of the included studies was evaluated by the same six authors, divided into the same three groups, using the Cochrane risk‐of‐bias assessment.^25^ The assessment evaluates the risk of bias of RCT in seven domains: (i) random sequence generation; (ii) allocation concealment; (iii) blinding of participants and personnel; (iv) blinding of outcome assessment; (v) incomplete outcome data; (vi) selective outcome reporting; and (vii) other sources of bias. The rating for each domain can be *yes* (low risk of bias), *no* (high risk of bias), or *unclear* (unclear risk). Any disagreement was resolved by systematic and thorough discussions with author Y. T.

### Statistical analyses

We used the Cochrane Collaboration Review Manager software (RevMan 5.3, Nordic Cochrane Centre, Cochrane Collaboration, Copenhagen, Denmark) for statistical analysis. Continuous outcome data from intervention and control groups were analyzed using effect sizes (standardized mean differences) with 95% confidence intervals (CI). Dichotomous outcomes were analyzed using risk ratios (RR) with 95%CI. We used random effects models in all analyses. Publication bias was evaluated by a funnel plot of treatment effect against standard error.^25^ Assessments of therapeutic effects and adverse events were reviewed. We also conducted a subgroup analysis to investigate sources of heterogeneity. We investigated: (i) whether trials with higher intensities of light (>5000 lx) had a higher antidepressant effect than trials with lower intensities of light (≤5000 lx); (ii) whether blue light, which maximally activates the region of the brain involved in emotional processing, has a higher antidepressant effect than white light^26^; (iii) whether trials with shorter intervention durations (≤2 weeks) had a higher antidepressant effect than trials with longer intervention durations (>2 weeks), which has been reported for nonseasonal depression^27^, ^28^; and (iv) whether trials with BLT administered in the morning differed from trials with BLT administered at other times because, while there is no direct comparison between morning and non‐morning BLT for BD,^29^ the efficacy of morning BLT for seasonal affective disorder is supported by evidence.^30^, ^31^

We also conducted a sensitivity analysis on primary outcome remission rates from depressive episodes in studies with a low overall indirectness because indirectness contributed to the marked heterogeneity in the analysis of the primary outcome.

## Results

### Description of studies included in the review

**Figure 1:**
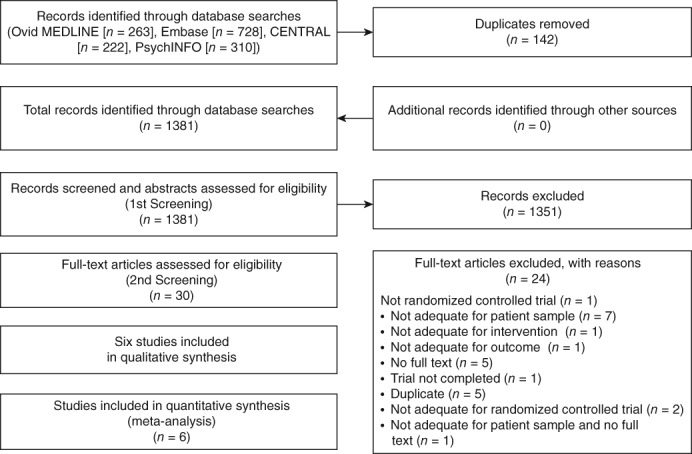
Flowchart of the study selection process. CENTRAL, Cochrane Central Register of Controlled Trials.

The initial literature search yielded 1381 unique entries published up to June 2019 (Ovid MEDLINE = 263, Embase = 728, CENTRAL = 222, PsychINFO = 310). Five ongoing clinical trials were identified on http://clinicaltrails.gov up to June 2019, but data were only available for some of these trials (two trials: not sure if completed; one trial completed; one trial terminated; one trial ongoing; see Table S2). After screening the titles and abstracts of the identified reports, the full‐text versions of a total of 30 studies were reviewed. Twenty‐four studies were excluded due to various reasons (see Table S3) and six studies remained. All of the remaining six RCT (Fig. 1) investigated antidepressant effects of BLT in bipolar depression. No study investigated the effect of BLT in preventing the recurrence of mood episodes in the neutral state or in improving manic symptoms in the manic state.

### Study characteristics

Six articles consisting of six studies published between 2000 and 2018 were included in this review.^17^, ^18^, ^20^, ^32^, ^33^, ^34^ Sample size ranged from 27 to 80, with a total of 297 participants (Tables 1 and S4). Of all participants, 62.0% were female and the mean age was 42.1 years. The criteria used for the diagnosis of bipolar depression varied across studies. Five studies used the DSM,^17^, ^20^, ^32^, ^33^, ^34^ and one study used clinical criteria.^18^ The numbers of participants taking medications also varied across studies. Mood stabilizers were used by 40.7% to 100% of participants, antipsychotics by 42.5% to 100%, and antidepressants by 0% to 100%.

**Table 1:** Characteristics of the study participants

Study (year)	Intervention/Control (*n*)	Age (years)	Female	Type of BD (I/II)	Onset age (years)	Number of mood episodes	Seasonality	Taking mood stabilizer (%)	Taking AP (%)	Taking AD (%)
Yorguner Küpeli *et al*. (2018)^32^	Intervention (16)	42.1 (9.1)	10/16 (62.5%)	I: 10/16 (62.5%) II: 6/16 (37.5%)	25.81 (5.84)	NA	4/16 (25.0%)	Li 9/16 (56.2%) LTG 2/16 (12.5%) VPA 3/16 (18.8%) CBZ 0/16 (0%)	6/16 (37.5%)	6/16 (37.5%)
	Control (16)	37.1 (8.2)	16/16 (100%)	I: 7/16 (43.8%) II: 9/16 (56.2%)	22.62 (5.36)	NA	3/16 (18.8%)	Li 8/16 (50.0%) LTG 5/16 (31.3%) VPA 3/16 (18.8%) CBZ 2/16 (12.5%)	9/16 (56%)	6/16 (37.5%)
Colombo *et al*. (2000)^34^	Intervention (42)	44.0 (12.4)	29/40 (72.5%)	NA	28.2 (11.8)	7.5 (5.4)	NA	Li 17/40 (42.5%)	NA	NA
	Control (38)	43.3 (13.6)	22/33 (66.7%)	NA	28.1 (8.70)	9.2 (10.1)	NA	Li 14/33 (42.4%)	NA	NA
Sit *et al*. (2018)^17^	Intervention (23)	45.7 (14.3)	60.9% (14/23)	I: 13/23 (56.5%) II: 10/23 (43.5%)	16.8 (8.5)	NA	19/23 (82.6%)	AC 12/23 (52.2%) Li 6/23 (26.1%)	14/23 (60.9%)	17/23 (73.9%)
	Control (23)	43.7 (15.0)	73.9% (17/23)	I: 18/23 (78.3%) II: 5//23 (21.7%)	15.3 (8.9)	NA	19/23 (82.6%)	AC 15/23 (65.2%) Li 4/23 (17.4%)	17/23 (73.9%)	19/23 (82.6%)
Dauphinais *et al*. (2012)^20^	Intervention (18)	42.4 (12.4)	72.2% (13/18)	NA	NA	NA	NA	NA	NA	NA
	Control (20)	43.1 (16.0)	75% (15/20)	NA	NA	NA	NA	NA	NA	NA
Zhou *et al*. (2018)^18^	Intervention (37)	35.1 (14.2)	60.6% (20/33)	NA	NA	NA	NA	100% (37/37)	100% (37/37)	0% (0/37)
	Control (37)	39.7 (13.5)	46.7% (14/30)	NA	NA	NA	NA	100% (37/37)	100% (37/37)	0% (0/37)
Franchini *et al*. (2009)^33^	Intervention (17)	45.2 (14.9)	41.2% (7/17)	NA	32.8 (13.2)	6.6 (4.5)	NA	Li 5/17 (29.4%)	NA	17/17 (100%)
	Control (10)	54.0 (12.2)	70.0% (7/10)	NA	41.3 (12.0)	8.5 (3.5)	NA	Li 6/10 (60.0%)	NA	10/10 (100%)

AC, anticonvulsants; AD, antidepressants; AP, antipsychotics; BD, bipolar disorder; CBZ, carbamazepine; Li, lithium; LTG, lamotrigine; NA, not available; VPA, valproate.

All studies were individual RCT.^17^, ^18^, ^20^, ^32^, ^33^, ^34^ Five studies were conducted at a secondary‐care facility^17^, ^18^, ^32^, ^33^, ^34^ while the level of care of one study was unknown.^20^ Four studies received financial support not related to BLT,^17^, ^18^, ^20^, ^33^ one study did not receive any financial support,^32^ and one study did not reveal financial support.^34^ There were four two‐arm studies^17^, ^18^, ^32^, ^33^ and two three‐arm studies.^20^, ^34^

Interventions (Tables 2 and S5) varied regarding light intensities (i.e., 10 000, 7000, 5000, or 2500 lx), light colors (i.e., 10 000 or 4000 K), exposure times (i.e., 7.5–45, 15–60, 30, or 60 min), intervention durations (i.e., 6 days, 2 weeks, 6 weeks, 8 weeks), and intervention timing (04.45–08.45 hours, 06.30–09.00 hours, 08.00–10.00 hours, approximately 08.00–09.00 hours, 12.00–14.30 hours, and ‘morning’ without giving a precise time). The control interventions also varied regarding light intensities. For example, four studies used dim light (less than 500, 150, less than 100, or 50 lx) while one study used low‐density negative air ionization, and one study used treatment as usual in the control group.

**Table 2:** Details of the interventions

Study (year)		Intervention	Intensity, color, and duration of each intervention session	Numbers and duration of intervention	Timing of intervention
Yorguner Küpeli *et al*. (2018)^32^	Intervention (16)	BLT	10 000 lx, 30 min, color: NA	2 weeks	08.00–10.00 hours
	Control (16)	Dim light	Less than 500 lx, 30 min, color: NA		
Colombo *et al*. (2000)^34^	Intervention (42)	BLT and TSD	2500 lx, 30 min, white light	6 days	03.00 hours during the TSD night, in the morning after the recovery sleep, and half an hour after awakening, between approximately 08.00 hours and 09.00 hours
	Control (38)	Dim light and SD	150 lx, 30 min, red light		
Sit *et al*. (2018)^17^	Intervention (23)	BLT	7000 lx, 15–60 min, white light (4000 K)	6 weeks	12.00–14.30 hours
	Control (23)	Dim light	50 lx, 15–60 min, red light		
Dauphinais *et al*. (2012)^20^	Intervention (18)	BLT	7000 lx, 7.5–45 min, white light (4000 K)	8 weeks	In the morning
	Control (20)	LDA	NA		
Zhou *et al*. (2018)^18^	Intervention (37)	BLT	5000 lx, 60 min, blue (10 000 K)	2 weeks	06.30–09.00 hours
	Control (37)	Dim light	Less than 100 lx, 60 min, Color: red		
Franchini *et al*. (2009)^33^	Intervention (17)	BLT + fluvoxamine	10 000 lx, 30 min, color: NA	From day 2 to the end of week 6	Between 04.45– 08.45 hours, depending on individual chronotype, measured by MEQ
	Control (10)	Fluvoxamine alone	Not applicable		Not applicable

BLT, bright light therapy; LDA, low‐density negative air ionization; MEQ, Morningness–Eveningness Questionnaire; NA, not available; TSD, total sleep deprivation.

### Risk‐of‐bias assessment

**Figure 2:**
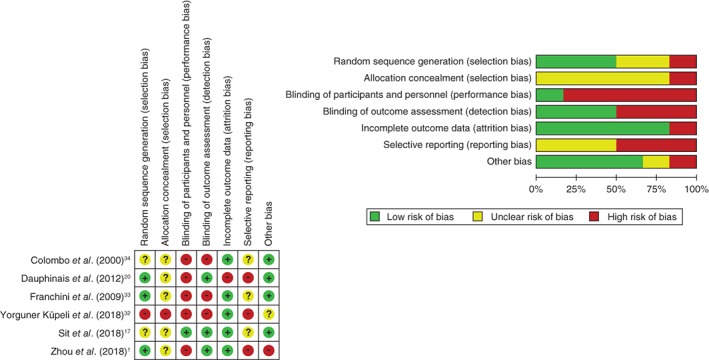
Risk‐of‐bias assessment. Green indicates a low risk of bias, yellow an unclear risk of bias, and red indicates a high risk of bias.

The risk‐of‐bias evaluation (Fig. 2) showed the following: three RCT had adequate randomization methods^18^, ^20^, ^33^; no RCT reported a sufficient allocation concealment procedure; five RCT had a high risk of bias in the participant and personnel blinding domain^18^, ^20^, ^32^, ^33^, ^34^; and three RCT were judged to have a high risk of bias in the blinding of outcome assessments domain.^32^, ^33^, ^34^ With regard to incomplete outcome data, one RCT had a high risk of bias due to a high dropout rate.^20^ Three studies had an unclear reporting bias (one study: unable to obtain the research registration^33^, ^34^; one study: endpoint timing was different from the protocol^17^). The other three studies had a high risk of reporting bias.^18^, ^20^, ^32^

### Treatment outcome assessment

Outcomes are summarized in Tables 3 and S6. Three RCT reported remission rates from depressive episodes after BLT as the primary study outcome.^17^, ^20^, ^32^ Remission rates from depressive episodes in two other studies were calculated by using depressive symptom scores.^18^, ^33^ Scores of depressive symptoms (SIGH‐ADS = 2, HAM‐D_17_ = 1, HAM‐D_21_ = 1) were reported in four studies.^17^, ^18^, ^20^, ^33^ All six RCT assessed manic switching rates during the study period (rating scale: one study; observation or not described: five studies).^17^, ^18^, ^20^, ^32^, ^33^, ^34^ One study evaluated daytime dysfunction using the Global Assessment of Functioning Scale.^17^ Two studies evaluated sleep quality using the Pittsburgh Sleep Quality Index.^17^, ^32^ Four studies reported adverse events as a result of the intervention.^17^, ^18^, ^20^, ^32^ Attrition was 14.1% (42/297).

**Table 3:** Summary of the outcomes

Study (year)	Study design, blinding	Measure for depressive symptoms (definition of remission from depressive episode)	Remission rate from depressive episode post‐intervention	Measure for manic symptoms (definition of manic switching)	Manic switching from depressive phase	Attrition
Yorguner Küpeli *et al*. (2018)^32^	RCT (two‐arm) Single blind (participants)	1) HAM‐D_17_ (≤7) 2) MADRS 10 items (≤9) 3) SIGH‐SAD 29 questions (ND)	At 2 weeks: 1) 44% (7/16) vs 6% (1/16)* 2) 44% (7/16) vs 12.5% (2/16) *	Observation	At 2 weeks: 0%	32/32 =100%
Colombo *et al*. (2000)^34^	RCT (three‐arm) Open labeled	1) Mean VAS† (ND) 2) HAM‐D_21_ ‡ (ND)	NA	Observation	At day 1–7: 4.8% (2/42) vs 13.2% (5/38)	73/80 =91.3%
Sit *et al*. (2018)^17^	RCT (two‐arm) Double blind (participants and assessor)	1) SIGH‐ADS (≤8) 2) HAM‐D_21_ ‡ (ND)	At 4–6 weeks: 1) 68.2% (15/22) vs 22.2% (4/18)*	YMRS (≥5)	At 1–6 weeks: 0% (0/22) vs 0% (0/18)	40/46 =87.0%
Dauphinais *et al*. (2012)^20^	RCT (three‐arm) Single blind (assessor)	1) SIGH‐ADS (≤8) 29 item 2) MADRS (ND) 10 item	1) 2/18(11.1%) vs 5/20 (25.0%)	YMRS (ND)	At 1–8 weeks: 4/18 (22.2%) vs 2/20 (10%)	21/38 =55.3%
Zhou *et al*. (2018)^18^	RCT (two‐arm) Single blind (participants)	1) HAM‐D17 (≤7) 2) QIDS‐SR 16 (ND)	At week 2: 1) 11/33 (33.3%) vs 4/30 (13.3%)	YMRS (ND)	At 1–2 weeks: 0/33 (0%) vs 0/30 (0%)	63/74 =85.1%
Franchini *et al*. (2009)^33^	RCT (two‐arm) Open labeled	1) HAM‐D_21_ ‡ (≤8)	At week 6: 1) 16/16 (100%) vs 10/10 (100%)	Observation	At 1–6 weeks: 1/17 (5.9%) vs 0/10 (0%)	26/27 =96.3%

* Significant difference (*P* < 0.05).

† Mean VAS: 08:00, 13:00, 18:00.

‡ Only after run‐in period.

HAM‐D_17_, 17‐item Hamilton Depression Rating Scale; HAM‐D_21_, 21‐item Hamilton Depression Rating Scale; MADRS, Montgomery–Åsberg Depression Rating Scale; NA, not available; ND, not defined; VAS, Visual Analog Scale; QIDS‐SR, 16‐item Quick Inventory of Depressive Symptomatology Self‐Report; RCT, randomized controlled trial; SIGH‐ADS, Structured Interview Guide for the Hamilton Depression Rating Scale With Atypical Depression Supplement; SIGH‐SAD, Structured Interview Guide for the Hamilton Depression Rating Scale With Seasonal Affective Disorder Supplement; YMRS, Young Mania Rating Scale.

**Figure 3:**
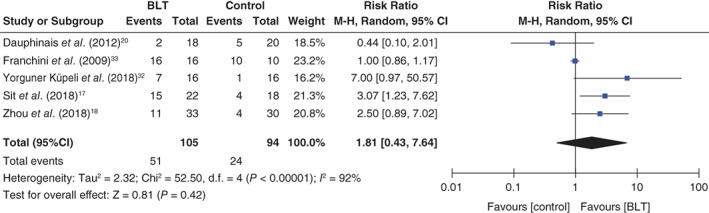
Forest plot of post‐intervention treatment effect sizes for remission rates from depressive episodes. BLT, bright light therapy; CI, confidence interval; SD, standard deviation.

**Figure 4:**
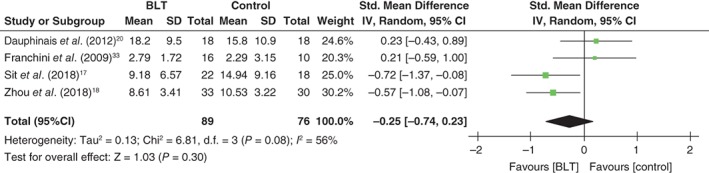
Forest plot of post‐intervention treatment effect sizes for scores of depressive symptoms. BLT, bright light therapy; CI, confidence interval; SD, standard deviation.

**Figure 5:**
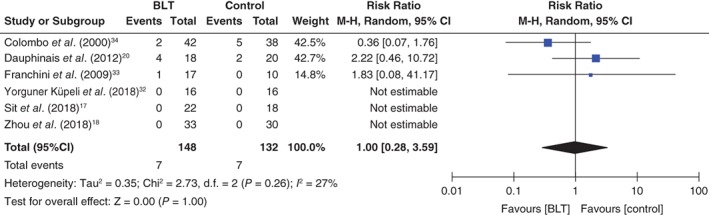
Forest plot of post‐intervention treatment effect sizes for manic switching from depressive episodes. BLT, bright light therapy; CI, confidence interval; SD, standard deviation.

There were no significant differences between the intervention and control groups in remission rates from depressive episodes (RR: 1.81, 95%CI: 0.43 to 7.64, *P* = 0.42, 199 participants, five studies; Fig. 3) or in scores for depressive symptoms (standardized mean difference: −0.25, 95%CI: −0.74 to 0.23, *P* = 0.30, 165 participants, four studies; Fig. 4). There was also no significant difference in manic switching between the two groups (RR: 1.00, 95%CI: 0.28 to 3.59, *P* = 0.26, 280 participants, six studies; Fig. 5). No meta‐analysis was performed on sleep quality and daytime functioning because only one study reported these two outcome measurements. Sit *et al*. reported significantly better daytime functioning in the BLT group than in the control group (Global Assessment of Functioning Scale score 74.77 ± 9.70 vs 67.65 ± 9.86, respectively; β = 7.13, *P* = 0.030; adjusted β = 7.61, *P* = 0.042), but no significantly better sleep quality in the BLT group (Pittsburgh Sleep Quality Index 5.80 ± 3.25 vs 6.19 ± 3.35, respectively; β = −0.39, *P* = 0.728; adjusted β = −0.93, *P* = 0.380). Suicidality was not reported in any study. Four studies examined differences in adverse events between groups using a standardized method.^17^, ^18^, ^20^, ^32^ Three of these studies reported details about adverse events^18^, ^20^, ^32^ but found no significant difference between the groups.^18^, ^20^, ^32^ Sit *et al*. reported that the BLT group had less excessive sleepiness as well as less trouble concentrating than the control group, but found no difference in suicide ideation between the two groups.^17^ The following detailed adverse events associated with BLT were reported: headache = 4.7% (7/148), sleep disturbance = 1.4% (2/148), insomnia = 0.68% (1/148), eyestrain = 0.68% (1/148), fatigue = 0.68% (1/148), dizziness = 0.68% (1/148), and state of confusion and sedation = 0.68% (1/148). For the control group, the following adverse events were reported: headache = 4.5% (6/132), insomnia = 0.76% (1/132), nightmares = 0.76% (1/132), nausea = 0.76% (1/132), palpitation = 0.76% (1/132), and irritability = 0.76% (1/132).^18^, ^20^, ^32^ Two studies did not report any adverse events other than manic switching.^33^, ^34^

The sensitivity analyses showed a significant difference in remission rates from depressive episodes between the BLT and control groups (RR: 3.09, 95%CI: 1.62 to 5.90, *P* = 0.006, 94 participants, three studies^17^, ^18^, ^32^) with low heterogeneity (*I* ^2^: 0%; Fig. S1). The subgroup analyses showed no significant differences in remission rates from depressive episodes between different light intensities (≤5000 lx [18] vs >5000 lx^17^, ^20^, ^32^, ^33^; Fig. S2) or colors (blue^18^ vs white^17^, ^20^; Fig. S3). However, there was a significant difference in remission rates from depressive episodes for the total duration of therapy (≤2 weeks [RR: 3.12, 95%CI: 1.25 to 7.78, *P* = 0.01, two studies^18^, ^32^] vs >2 weeks [RR: 1.18, 95%CI: 0.31 to 4.50, *P* = 0.81, three studies^17^, ^20^, ^33^]; Fig. S4) and for the timing of light exposure (morning [g = 1.56, 95%CI: 0.35 to 6.97, *P* = 0.56, four studies^18^, ^20^, ^32^, ^33^] vs other times [g = 3.07, 95%CI: 1.23 to 7.62, *P* = 0.02, one study^17^]; Fig. S5).

## Discussion

To our knowledge, this is the first systematic review and meta‐analysis that has evaluated RCT investigating the efficacy of BLT in BD. The results of this review do not show an effect of BLT on bipolar depression. Additionally, our analyses indicate that BLT does not increase the risk of manic switching in patients with BD. None of the studies included in our analysis reported serious adverse events during the study period. We were unable to perform a meta‐analysis on the efficacy of BLT for bipolar mania or its effect in preventing recurrent mood episodes in the euthymic state because none of the included RCT examined these aspects.

The results of this study, which suggest that BLT does not have a significant antidepressant effect, are inconsistent with those of a previous meta‐analysis by Tseng *et al*. and a recent systematic review by the ISBD Task Force, which suggested that BLT is an effective treatment for bipolar depression.^19^, ^21^ Among the 11 studies^20^, ^22^, ^34^, ^35^, ^36^, ^37^, ^38^, ^39^, ^40^, ^41^, ^42^ included in the meta‐analysis by Tseng *et al*.,^21^ seven were not RCT and had a single‐arm design^35^, ^36^, ^38^, ^39^, ^40^, ^41^, ^42^ and six studies combined BLT and SD without an SD‐only control group.^35^, ^36^, ^37^, ^38^, ^40^, ^41^ Moreover, 70% of the subjects in one study had major depressive disorder,^22^ which might have affected the results. The systematic review conducted by the ISBD Task Force also included non‐RCT, and their recommendations were derived from the consensus opinions of the authors, which were based on a synthesis of the efficacy and tolerability data generated from their review.^19^ In contrast, our meta‐analysis evaluated only RCT and only trials with at least 80% of patients with BD. We also made sure that the control group treatment conditions were similar to those of the intervention group. Our analysis therefore has a more accurate effect size than the previous meta‐analysis. Differences in the systematic review methods between this study and previous studies may have led to differing results. Although some well‐designed RCT showed statistically significant therapeutic effects of BLT on depressive symptoms, the present meta‐analysis found a lack of significant effect compared to controls. This inconsistency could be due to the high heterogeneity of the interventions and control conditions across studies. Additionally, our sensitivity analysis for studies with low overall indirectness did show a significant antidepressant effect of BD, as well as minimal heterogeneity between groups. The result of our sensitivity analysis was consistent with the ISBD Task Force's recommendations.^19^ The lack of a significant antidepressant effect in our review may stem from the inclusion of only RCT, as well as the small sample sizes and high heterogeneity of the studies. Additional well‐designed studies will be required in order to conclusively demonstrate the potential of BLT as an effective chronotherapeutic treatment option for bipolar depression.

Our results do not allow us to conclude that there is a lack of effect of BLT on bipolar depression, as the meta‐analysis was based on a small number of RCT that were highly heterogeneous. Indeed, the sensitivity analysis for three RCT with low indirectness did show an effect of BLT on bipolar depression. It would be appropriate to judge the usefulness of BLT for the treatment of bipolar depression by considering not only RCT but also case series and expert opinions based on existing evidence. The current systematic review highlights the lack of available high‐quality RCT that can inform the clinical practice.

Several other factors, such as the specific study design, what BLT was compared to (dim light or others), or concomitant pharmacological treatment, may have led to the conflicting results between our and earlier studies. Another source of inconsistencies is the heterogeneity of subject characteristics across different RCT. For example, the study conducted by Sit *et al*. included 82.6% of patients with BD with characteristics of seasonality.^17^ As BLT has been reported to be more effective for seasonal depression than for nonseasonal depression, due to its connection with the circadian rhythm,^26^, ^43^ it is not surprising that BLT is more effective for BD with seasonal characteristics. Differences in the severity of depressive symptoms at baseline may also have affected results. For example, Franchini *et al*. enrolled subjects who had severe BD with psychotic features, with a mean HAM‐D_21_ of 30 and more,^33^ whereas four other studies enrolled subjects with only moderate BD with a mean HAM‐D_21_ in the 20s’ range. Terman *et al*. reported that BLT is more effective for seasonal affective disorder with fewer depressive symptoms.^43^ Specific details of the light therapy, such as light intensity, light color, duration of exposure, or exposure timing, may also have affected results. Our subgroup analysis showed a significant effect for shorter durations of light and for exposure during daytime. Additional studies are needed to investigate the most appropriate method to implement BLT in the treatment of bipolar depression. Last but not least, a responder analysis is warranted to identify subgroups of bipolar depression for which BLT is more effective. BLT will be a more useful treatment for bipolar depression if predictors of treatment response to BLT are identified.

As an alternative chronotherapy, SD is also used for bipolar depression, and has been recommended by the ISBD Task Force.^19^ Its effectiveness has also been demonstrated by a meta‐analysis.^44^ Furthermore, BLT may be performed in combination with SD to enhance and maintain the effect of SD.^35^, ^45^ Thus, the combination of different chronotherapies is a viable treatment option. However, RCT that have investigated the effect of BLT on SD are limited,^34^ and further studies are required.

In regard to manic switching, the results of our study indicate no significant differences in manic switching rates between the BLT and control groups. This is in line with a previous meta‐analysis published in 2016.^21^ In our study, the manic switching rate was 4.7% in the BLT group, higher than the 2.3% (18/799) reported in a previous historical review.^46^ Rates of manic switching also depended on the evaluation method (rating scale: 3%, clinical mental state examination: 0.8%, no method reported: 0%).^46^ In our study, 27% (40/148) of patients were evaluated for manic switching using a rating scale, a higher rate than the 12.5% (100/799) of patients evaluated in the previous review, which probably led to higher detection rates of manic switching. Furthermore, concurrent use of mood stabilizers with BLT might affect manic switching rates. Antidepressant monotherapy increases manic switching,^47^ whereas a combination of antidepressants and mood stabilizers does not.^48^ As BLT is believed to have the same mechanism of enhancing serotonin levels that most antidepressants have,^49^, ^50^ BLT without mood stabilizers may increase manic switching. Regarding other adverse events, previous studies have generally determined that BLT is a noninvasive therapy for treating monopolar or bipolar depression.^15^, ^21^, ^27^, ^28^, ^30^, ^51^, ^52^, ^53^ Based on the RCT included in this review, we also conclude that BLT is a safe treatment for BD within the period investigated (i.e., 8 weeks).

Sleep quality and daytime functioning were not analyzed, because only one study reported on these measures. As modulating circadian rhythms could be an underlying mechanism of BLT, it is possible that BLT improves sleep symptoms, which eventually leads to improved mood. However, Sit *et al*. reported no significant difference in sleep quality, in spite of a significant difference in remission rates from depressive episodes,^17^ suggesting that BLT can improve depressive moods without improving sleep. BLT could improve depression not only indirectly by regulating sleep and circadian rhythms but also directly by regulating mood states.^26^ The findings of Sit *et al*. seem to support the possibility of a direct pathway mechanism underlying the effect of BLT on depressive symptoms.

Our study has several limitations. First, we only included six RCT with a total of 280 subjects, leading to relatively low statistical power. Further RCT with more participants are therefore needed to clarify the effectiveness of BLT for bipolar depression. Second, specific parameters of BLT, such as light intensity, exposure duration, and color and temperature of the light, differed across the studies included in our meta‐analysis, which might have led to the high heterogeneity of our results. Well‐designed RCT are needed to examine the best protocol for BLT in the treatment of bipolar depression. Third, this study only included short‐term RCT with a duration of 8 weeks or less. The long‐term efficacy of BLT for bipolar depression is therefore still unclear. To investigate the appropriate duration, long‐term observational studies are warranted. Finally, indications of BLT in BD other than depressive states are still unknown (manic, mixed, or euthymic), due to the lack of studies investigating the efficacy of BLT in the prevention of recurrence from euthymic states or its therapeutic effect on mania phases or mixed states. Further studies are thus needed to investigate the comprehensive efficacy and safety of BLT for patients with BD.

In conclusion, the results of our meta‐analysis of RCT suggest that BLT does not significantly improve depressive symptoms in BD, and does not increase the risk of manic switching in patients with bipolar depression. Nevertheless, the results do not conclusively demonstrate the lack of an effect of BLT on bipolar depression, as our meta‐analysis was based on a small number of RCT that were highly heterogeneous. Further studies with larger sample sizes and appropriate control groups are necessary to draw definitive conclusions regarding the efficacy and safety of BLT for bipolar depression.

## Disclosure statement

Yumi Aoki and Zhe Wang declare no conflict of interest. Masahiro Takeshima has received lecture fees from Daiichi Sankyo Company. Tomohiro Utsumi has received lecture fees from Eisai. Masahiro Suzuki has received speaker's honoraria from Dainippon Sumitomo Pharma, Eli Lilly, Eisai, Meiji Seika Pharma, MSD, Otsuka Pharmaceutical, Pfizer, and Takeda Pharmaceutical, and research funding from Novartis. Isa Okajima has received lecture fees from Otsuka Pharmaceutical, MSD, and Takeda Pharmaceutical, and research funding from NEC Solution Innovators. Norio Watanabe has received royalties from Sogensha, Medical Review, and Akatsuki. Koichiro Watanabe has received manuscript fees or speaker's honoraria from Daiichi Sankyo, Eisai, Eli Lilly, GlaxoSmithKline, Janssen Pharmaceutical, Kyowa Pharmaceutical, Meiji Seika Pharma, Mitsubishi Tanabe Pharma, MSD, Otsuka Pharmaceutical, Pfizer, Shionogi, Sumitomo Dainippon Pharma, Takeda Pharmaceutical, and Yoshitomi, and research/grant support from Astellas Pharma, Daiichi Sankyo, Eisai, MSD, Mitsubishi Tanabe Pharma, Meiji Seika Pharma, Otsuka Pharmaceutical, Pfizer, Shionogi, and Sumitomo Dainippon Pharma. He is also a consultant for Eisai, Eli Lilly, Kyowa Pharmaceutical, Otsuka Pharmaceutical, Pfizer, Sumitomo Dainippon Pharma, Taisho Toyama Pharmaceutical, and Takeda Pharmaceutical. Yoshikazu Takaesu has received lecture fees from Otsuka Pharmaceutical, Meiji Seika Pharma, Eli Lilly, Eisai, Mitsubishi Tanabe Pharma, MSD, and Yoshitomi Pharmaceutical, and research funding from Otsuka Pharmaceutical, Meiji Seika Pharma, MSD, and Eisai.

## Author contributions

M.T. was responsible for the literature screening and wrote the Abstract, Methods, Results, and Discussion of this manuscript. T.U. was involved in the literature screening and statistical procedures. Y.A. was involved in the literature search and screening. Z.W. wrote the Introduction and was involved in the literature search and screening. M.S. helped with the presentation of the current evidence on BLT for BD and took part in the literature screening. I.O. took part in the literature screening. N.W. provided important comments on possible confounding factors and the direction of the current meta‐analysis. K.W. provided excellent comments on the entire manuscript. Y.T., the corresponding author, takes responsibility for collecting all information, for implementing all ideas contributed by the other authors, and for the final revision and submission of the manuscript.

## Supporting information


**Figure S1.** Sensitivity analysis of the studies with low indirectness.

**Figure S2.** Subgroup analysis of light intensities.

**Figure S3.** Subgroup analysis of light colors.

**Figure S4.** Subgroup analysis of treatment durations.

**Figure S5.** Subgroup analysis of timing of light exposure.

**Table S1.** Search strategies.

**Table S2.** List of the trials on http://clincaltrials.gov.

**Table S3.** List of excluded articles.

**Table S4.** Complete characteristics of all study participants.

**Table S5.** Complete details of all interventions.

**Table S6.** Complete summary of outcomes.
