# Supplementary material for: Efficacy and safety of bright light therapy for manic and depressive symptoms in patients with bipolar disorder: A systematic review and meta‐analysis
Source: Psychiatry Clin Neurosci. 2020 Feb 10;74(4):247–56. doi: 10.1111/pcn.12976 (PMC7187384; doi:10.1111/pcn.12976)
Supplement: Supplementary file 6 — Table S1. Search strategies. [file PCN-74-247-s006.docx]

**Supporting Table S1. Search strategies**

We searched the databases OvidMedline, CENTRAL, Embase, and PsychINFO using the following search terms:

**OvidMedline**

1 　 exp Bipolar Disorder/

2 　(Bipolar or cyclothymi$).ti,ab,kw,ot.

3 　 ((Mani$ or major) and (depres$ or disorder$)).ti,ab,kw.

4 　 1 or 2 or 3

5 　exp Phototherapy/

6 　 “Color Therap$” {Including Related Terms}

7 　Heliotherap$ {Including Related Terms}

8 　“Intense Pulsed Light Therap$” {Including Related Terms}

9 　“Low Level Light Therap$” {Including Related Terms}

10　 “Low-Level Light Therap$” {Including Related Terms}

11　 exp Low-Level Light Therapy/

12　 exp Low Level Light Therapy/

13　 Photochemotherap$ {Including Related Terms}

14 　“Hematoporphyrin Photoradiation” {Including Related Terms}

15　 “Ultraviolet Therap$” {Including Related Terms}

16　 “PUVA Therap$” {Including Related Terms}

17 　Photopheresis {Including Related Terms}

18 5 or 6 or 7 or 8 or 9 or 10 or 11 or 12 or 13 or 14 or 15 or 16 or 17

19 4 and 18

20 randomized controlled trial.pt.

21 controlled clinical trial.pt.

22 randomized.ab.

23 placebo.ab.

24 drug therapy.fs.

25 randomly.ab.

26 trial.ab.

27 groups.ab.

28 20 or 21 or 22 or 23 or 24 or 25 or 26 or 27

29 exp animals/ not humans.sh.

30 28 not 29

31 19 and 30

**CENTRAL**

1. ("bipolar disorder")
2. (Bipolar or cyclothymi$)
3. ((Mani$ or major) and (depres$ or disorder$))
4. 1 OR 2 OR 3
5. Phototherapy
6. “Color Therap$”
7. “Intense Pulsed Light Therap$”
8. “Low Level Light Therap$”
9. “Low-Level Light Therap$”
10. Photochemotherap$
11. “Hematoporphyrin Photoradiation”
12. “Ultraviolet Therap$”
13. “Ultraviolet Therap$”
14. “PUVA Therap$”
15. Photopheresis
16. 5 OR 6 OR 7 OR 8 OR 9 OR 10 OR 11 OR 12 OR 13 OR 14 OR 15
17. 4 AND 16

**Embase**

1. bipolar AND 'disorder'/exp
2. bipolar OR cyclothymi*
3. (mani* OR major) AND (depres* OR disorder*)
4. 1 OR 2 OR 3
5. phototherapy'/exp
6. color therap*'
7. heliotherap*
8. intense pulsed light therap*'
9. low level light therap*'
10. low-level light therap*'
11. low level' AND light AND 'therapy'/exp
12. photochemotherapy*
13. hematoporphyrin photoradiation'
14. ultraviolet therap*'
15. puva therap*'
16. photopheresis
17. 5 OR 6 OR 7 OR 8 OR 9 OR 10 OR 11 OR 12 OR 13 OR 14 OR 15 OR 16
18. crossover procedure':de OR 'double-blind procedure':de OR 'randomized controlled trial':de OR 'single-blind procedure':de OR random*:de,ab,ti OR factorial*:de,ab,ti OR crossover*:de,ab,ti OR ((cross NEXT/1 over*):de,ab,ti) OR placebo*:de,ab,ti OR ((doubl* NEAR/1 blind*):de,ab,ti) OR ((singl* NEAR/1 blind*):de,ab,ti) OR assign*:de,ab,ti OR allocat*:de,ab,ti OR volunteer*:de,ab,ti
19. 4 AND 17 AND 18

**PsychINFO**

1 　　TX "bipolar disorder"

2　　 TX bipolar

3 　　TX cyclothymi*

4 　　TX mani*

5 　　TX major

6 　　TX depres*

7 　　TX disorder*

8 　　4 OR 5

9 　　6 OR 7

10 　 8 AND 9

11 　 1 OR 2 OR 3 OR 10

12 　　TX phototherapy

13 　　TX 'color therap*'

14　　 TX heliotherap*

15 　　TX 'intense pulsed light therapy'

16　　 TX 'low level light therap*'

17　　 TX 'low-level light therap*'

18 　　TX 'low level' AND light AND 'therapy'

19 　　TX photochemotherap*

20 　　TX 'hematoporphyrin photoradiation'

21 　　TX 'photoradiation'

22 　　TX 'ultraviolet therap*'

23 　　TX 'puva therap*'

24 　　TX photopheresis

25 　　12 OR 13 OR 14 OR 15 OR 16 OR 17 OR 18 OR 19 OR 20 OR 21 OR 22 OR 23 OR 24

26 　　11 AND 25

27 　　Narrow by Methodology: - prospective study, Narrow by Methodology: - scientific simulation, Narrow by Methodology: - followup study, Narrow by Methodology: - treatment outcome, Narrow by Methodology: - clinical trial, Narrow by Methodology: - quantitative study, Narrow by Methodology: - empirical study

**ClicalTrials.gov**

1 　 Bipolar disorder [condition or disease]

2 　 Light [other terms]
