# Supplementary material for: Efficacy and safety of bright light therapy for manic and depressive symptoms in patients with bipolar disorder: A systematic review and meta‐analysis
Source: Psychiatry Clin Neurosci. 2020 Feb 10;74(4):247–56. doi: 10.1111/pcn.12976 (PMC7187384; doi:10.1111/pcn.12976)
Supplement: Supplementary file 7 — Table S2. List of the trials on http://clincaltrials.gov. [file PCN-74-247-s007.docx]

**Supporting Table S2. List of the trials on ClincalTrials.gov**

| Study title | Status and data collection |
| --- | --- |
| Neural and Visual Responses to Light in Bipolar Disorder: A Novel Putative Biomarker (NCT02048995) | Completed. Data not available. |
| Bright Light Therapy in the Treatment of Non-seasonal Bipolar Depression (NCT03396744) | Ongoing trial. Data not available. |
| Light-Therapy in the Treatment of the Acute Phase of the Bipolar Type II Depression (NCT00590265) | Unknown status. Data not available. |
| The Role of Dopamine Metabolism in the Antidepressant Effects of Sleep Deprivation and Sertraline in Depressed Patients (NCT00581009) | Unknown status. Data not available. |
| Chronotherapy Randomized Controlled Trial (NCT02176824) | Terminated. |
