# Supplementary material for: Efficacy and safety of bright light therapy for manic and depressive symptoms in patients with bipolar disorder: A systematic review and meta‐analysis
Source: Psychiatry Clin Neurosci. 2020 Feb 10;74(4):247–56. doi: 10.1111/pcn.12976 (PMC7187384; doi:10.1111/pcn.12976)
Supplement: Supplementary file 8 — Table S3. List of excluded articles. [file PCN-74-247-s008.docx]

**Supporting Table S3. List of excluded articles**

| Study | Reason for exclusion |
| --- | --- |
| 1. Benedetti 2001 | Not appropriate for patient outcome (symptoms of either depression or mania were not measured) |
| 1. Benedetti 2003 | Not appropriate for patient sample (less than 80% with BD) |
| 1. Beauchemin 1997 | Not appropriate for patient sample (less than 80% with BD) |
| 1. Bogen 2013 | Not appropriate for patient age (12-18) |
| 1. Bogen 2017 | Not appropriate for patient age (12-18) |
| 1. Chojnacka 2016 | Not appropriate for patient sample (less than 80% with BD) |
| 1. Chojnacka 2016 | Not appropriate for patient sample (less than 80% with BD); conference presentation |
| 1. Chojnacka 2016 | Duplicate of 7 |
| 1. Deltito 1991 | Not appropriate for patient sample (less than 80% with BD) |
| 1. Heim 1988 | Not appropriate for patient sample (less than 80% with BD) and control (active control); not RCT |
| 1. Kripke 1992 | Not appropriate for patient sample (less than 80% with BD) |
| 1. Leibenluft 1995 | Not RCT (observational study) |
| 1. Nct | Ongoing clinical trial |
| 1. Sep-Kowalikowa 2002 | Not appropriate for patient sample (less than 80% with BD); not RCT |
| 1. Sit 2013 | No full text (conference presentation) |
| 1. Sit 2015 | No full text (conference presentation) |
| 1. Sit 2017 | No full text (conference presentation) |
| 1. Sit 2017 | No full text (conference presentation) |
| 1. Sit 2018 | Duplicate of included article (Am J Psychiatry. 2018;175(2):131-139) |
| 1. Sit 2018 | Duplicate of included article (Am J Psychiatry. 2018;175(2):131-139) |
| 1. Wu 2009 | Not appropriate for intervention (BLT+SD+SPA) |
| 1. Zhou 2016 | No full text (conference presentation) |
| 1. Zhou 2017 | Duplicate of included article (J Affect Disord. 2018;227:90-96) |
| 1. Zhou 2018 | Duplicate of included article (J Affect Disord. 2018;227:90-96) |

BD=bipolar disorder, BLT=blight light therapy, RCT=randomized controlled trial, SD=sleep deprivation, SPA=sleep phase advance.
