# Supplementary material for: Efficacy and safety of bright light therapy for manic and depressive symptoms in patients with bipolar disorder: A systematic review and meta‐analysis
Source: Psychiatry Clin Neurosci. 2020 Feb 10;74(4):247–56. doi: 10.1111/pcn.12976 (PMC7187384; doi:10.1111/pcn.12976)
Supplement: Supplementary file 9 — Table S4. Complete characteristics of all study participants. [file PCN-74-247-s009.docx]

**Supporting Table S4.** **Complete characteristics of all study participants**

| Study (year) | Intervention/Control (n) | Age | Female | Higher education (%) | Employed (%) | Married or cohabitant (%) | Type of BD (I/II) | Onset age | Number of mood episodes | Family history of BD | Seasonality | Concurrent psychiatric disorders (%) | Taking mood stabilizer (%) | Taking AP (%) | Taking AD (%) | Country |
| --- | --- | --- | --- | --- | --- | --- | --- | --- | --- | --- | --- | --- | --- | --- | --- | --- |
| Kupeli et al. (2018) ^(32)^ | Intervention　(16) | 42.1 (9.1) | 10/16 (62.5%) | 7/16 (43.8%) | 8/16 (50.0%) | 10/16 (62.5%) | I: 10/16 (62.5%)  II: 6/16 (37.5%) | 25.81 (5.84) | NS | 8/16 (50.0%) | 4/16 (25.0%) | 8/16 (50.0%) | Li 9/16 (56.2%)  LTG 2/16 (12.5%)  VPA 3/16 (18.8%)  CBZ 0/16 (0%) | 6/16 (37.5%) | 6/16 (37.5%) | Turkey |
|  | Control (16) | 37.1 (8.2) | 16/16 (100%) | 6/16 (37.5%) | 11/16 (68.8%) | 8/16 (50.0%) | I: 7/16 (43.8%)  II: 9/16 (56.2%) | 22.62 (5.36) | NS | 10/16 (62.5%) | 3/16 (18.8%) | 10/16 (62.5%) | Li 8/16 (50.0%)  LTG 5/16 (31.3%)  VPA 3/16 (18.8%)  CBZ 2/16 (12.5%) | 9/16 (56%) | 6/16 (37.5%) |  |
| Colombo et al. (2000) ^(34)^ | Intervention　(42) | 44.0　(12.4) | 29/40　(72.5%) | NS | NS | NS | NS | 28.2 (11.8) | 7.5 (5.4) | NS | NS | 0/42 (0%) | Li 17/40 (42.5%) | NS | NS | Italy |
|  | Control (38) | 43.3 (13.6) | 22/33　(66.7%) | NS | NS | NS | NS | 28.1 (8.70) | 9.2 (10.1) | NS | NS | 0/38 (0%) | Li 14/33 (42.4%) | NS | NS |  |
| Sit et al. (2018) ^(17)^ | Intervention　(23) | 45.7 (14.3) | 60.9% (14/23) | 11/23 (47.8%) | 12/23 (52.2%) | NS | I: 13/23 (56.5%)  II: 10/23 (43.5%) | 16.8 (8.5) | NS | NS | 19/23 (82.6%) | 17/23 (73.9%) | AC 12/23 (52.2%)  Li　6/23 (26.1%) | 14/23 (60.9%) | 17/23 (73.9%) | USA |
|  | Control (23) | 43.7 (15.0) | 73.9% (17/23) | 7/23 (30.4%) | 6/23 (26.1%) | NS | I: 18/23 (78.3%)  II: 5//23 (21.7%) | 15.3 (8.9) | NS | NS | 19/23 (82.6%) | 18/23 (78.3%) | AC 15/23 (65.2%)  Li　4/23 (17.4%) | 17/23 (73.9%) | 19/23 (82.6%) |  |
| Dauphinais et al. (2012) ^(20)^ | Intervention　(18) | 42.4 (12.4) | 72.2% (13/18) | NS | NS | NS | NS | NS | NS | NS | NS | NS | NS | NS | NS | USA |
|  | Control (20) | 43.1 (16.0) | 75% (15/20) | NS | NS | NS | NS | NS | NS | NS | NS | NS | NS | NS | NS |  |
| Zhou et al. (2018)　^(18)^ | Intervention　(37) | 35.1 (14.2) | 60.6% (20/33) | 45.5% (15/33) | NS | 48.5% (16/33) | NS | NS | NS | NS | NS | 0% | 100% (37/37) | 100% (37/37) | 0% (0/37) | China |
|  | Control (37) | 39.7 (13.5) | 46.7% (14/30) | 23.3% (7/30) | NS | 60.0% (18/30) | NS | NS | NS | NS | NS | 0% | 100% (37/37) | 100% (37/37) | 0% (0/37) |  |
| Franchini (2009) ^(33)^ | Intervention　(17) | 45.2 (14.9) | 41.2%  (7/17) | NS | NS | NS | NS | 32.8 (13.2) | 6.6 (4.5) | 12/17 (70.6%) | NS | 0% | Li 5/17 (29.4%) | NS | 17/17 (100%) | Italy |
|  | Control (10) | 54.0 (12.2) | 70.0% (7/10) | NS | NS | NS | NS | 41.3 (12.0) | 8.5 (3.5) | 7/10 (70.0%) | NS | 0% | Li 6/10 (60.0%) | NS | 10/10 (100%) |  |

AC = anticonvulsants, AD = antidepressants, AP = antipsychotics, BD = bipolar disorder, CBZ = carbamazepine, Li = lithium, LTG = lamotrigine, VPA = valproate. Higher education = college or more.
