# Supplementary material for: Efficacy and safety of bright light therapy for manic and depressive symptoms in patients with bipolar disorder: A systematic review and meta‐analysis
Source: Psychiatry Clin Neurosci. 2020 Feb 10;74(4):247–56. doi: 10.1111/pcn.12976 (PMC7187384; doi:10.1111/pcn.12976)
Supplement: Supplementary file 10 — Table S5. Complete details of all interventions. [file PCN-74-247-s010.docx]

**Supporting Table S5. Complete details of all interventions**

| Study (year) |  | Intervention | Intensity, color, and duration of each intervention session | Numbers and duration of intervention | Timing of intervention | Quality assurance of intervention |
| --- | --- | --- | --- | --- | --- | --- |
| Kupeli et al. (2018) ^(32)^ | Intervention (16) | BLT | 10,000 lx, 30 min, Color: NS | 2 weeks | 8:00 – 10:00 am | Light therapy was carried out in two identical rooms with room lighting by default below 50 lx, and the intensity of light exposure from the devices were measured by lux meters. The same rooms were used throughout the study and the placement of the light boxes remained consistent throughout the study. |
|  | Control (16) | Dim light | Less than 500 lx, 30 min, Color: NS |  |  |  |
| Colombo et al. (2000) ^(34)^ | Intervention (42) | BLT and TSD | 2,500 lx, 30 min, white light | 6 days | 3:00 am during the TSD night, in the morning after the recovery sleep, and half an hour after awakening, between approximately 8.00 am and 9.00 am | NS |
|  | Control (38) | Dim light and SD | 150 lx, 30 min, red light |  |  |  |
| Sit et al. (2018) ^(17)^ | Intervention (23) | BLT | 7,000 lx, 15-60 min, white light (4,000 K) | 6 weeks | 12:00 – 2:30 pm | The unit conforms to stringent standards, including illumination of a broad visual field, lighting from above to avoid glare, and maximal ultraviolet filtration. Participants were provided with standardized instructions on the appropriate use of their light box: optimal placement of the unit on its desk stand 12 inches from the eyes, and facing the light box without directly staring at it during the daily sessions of light therapy. |
|  | Control (23) | Dim light | 50 lx, 15-60 min, red light |  |  |  |
| Dauphinais et al. (2012) ^(20)^ | Intervention (18) | BLT | 7,000 lx, 7.5-45 min, white light (4000 K) | 8 weeks | In the morning | Participants were told that both BLT and negative ion therapy have been found to be effective in treating some forms of depression, although this had not been well studied in bipolar depression. Subjects were instructed to place the box on a desk or tabletop at an angle of 15°, to adjust the height so the center of the box was at eye level, and to use the 7,000-lx setting with their face fully exposed without staring into the light. |
|  | Control (20) | LDA | NA |  |  |  |
| Zhou et al. (2018) ^(18)^ | Intervention (37) | BLT | 5,000 lx, 60 min, blue (10,000 K) | 2 weeks | 6:30 – 9:00 am | In order to balance expectations, participants were told that both BLT and dRLT have been found to be effective in treating some forms of depression, although this had not been well studied in bipolar depression. The study design was presented as a comparison of different light therapies. The heights of light boxes were adjusted to make sure the center of the box was at eye level. |
|  | Control (37) | Dim light | Less than 100 lx, 60 min, Color: red |  |  |  |
| Franchini et al. (2009) ^(33)^ | Intervention (17) | BLT＋Fluvoxamine | 10,000 lx, 30 min, color: NS | From day 2 to the end of week 6 | Between 4:45 – 8:45 am, depending on individual chronotype, measured by MEQ | Nursing stuff provided the correct administration of light therapy and guaranteed that patients observed the defined time of light administration. Light sessions took place in a dedicated room of the ward, appropriately equipped for the purpose. |
|  | Control (10) | Fluvoxamine alone | NA |  | NA | NA |

BLT = bright light therapy, MEQ = morningness-eveningness questionaire, dRLT = dim red light therapy, SD = total sleep deprivation, LDA = low-density negative air ionization.
