# Supplementary material for: Efficacy and safety of bright light therapy for manic and depressive symptoms in patients with bipolar disorder: A systematic review and meta‐analysis
Source: Psychiatry Clin Neurosci. 2020 Feb 10;74(4):247–56. doi: 10.1111/pcn.12976 (PMC7187384; doi:10.1111/pcn.12976)
Supplement: Supplementary file 11 — Table S6. Complete summary of outcomes. [file PCN-74-247-s011.docx]

**Supporting Table S6. Complete summary of outcomes**

| Study  (year) | Study design, blinding | Diagnostic criteria for bipolar depression | Measure for depressive symptoms (definition of remission from depressive episode) | Remission rate from depressive episode post intervention | Measure for manic symptoms (definition of manic switching) | Manic switching from depressive phase | Measure for daytime functioning | Improvement in daytime functioning | Measure for insomnia symptoms | Improvement in insomnia symptoms | Attrition |
| --- | --- | --- | --- | --- | --- | --- | --- | --- | --- | --- | --- |
| Kupeli et al.  (2018) ^(32)^ | RCT (two-arm)  Single blind (participants) | SCID-I | 1) HAM-D_17_ (≤7)  2) MADRS 10 items (≤ 9)  3) SIGH-SAD 29 questions (NS) | At 2 wk:  1) 44% (7/16) vs 6% (1/16) #  2) 44% (7/16) vs 12.5% (2/16) # | Observation (NS) | At 2 weeks: 0% | NA | NA | PSQI 24 questions | NS | 32/32  = 100% |
| Colombo et al. (2000) ^(34)^ | RCT (three-arm)  Open labeled | Semi-SCID-I | 1) mean VAS (NS)  2) HAM-D_21_ (NS) | NS | Observation (NS) | At day 1-7:  4.8% (2/42) vs 13.2% (5/38) | NS | NS | NS | NS | 73/80  = 91.3% |
| Sit et al. (2018) ^(17)^ | RCT (two-arm)  Double blind (participants and assessor) | SCID-I | 1) SIGH-ADS (≤8)  2) HAM-D_21_ (NS) | At 4-6 wk:  1) 68.2% (15/22) vs 22.2% (4/18) # | MRS (≥5) | At 1-6 weeks:  0% (0/22) vs 0% (0/18) | GAF | 74.77(9.7) vs 67.65(9.86) | PSQI | At 4-6 wk:  5.80 (3.25) vs 6.19 (3.35) | 40/46  = 87.0% |
| Dauphinais et al. (2012) ^(20)^ | RCT (three-arm)  Single blind (assessor) | DSM-IV | 1) SIGH-ADS (≤8) 29 item  2) MADRS (NS) 10 item | 1) 2/18(11.1%) vs 5/20(25.0%) | YMRS (NS) | At 1-8 weeks:  4/18 (22.2%)  vs  2/20 (10%) | Q-LES-Q | NS | NS | NS | 21/38  = 55.3% |
| Zhou et al. (2018) ^(18)^ | RCT (two-arm)  Single blind (participants) | Clinical criteria | 1) HAM-D17 (≤7)  2) QIDS-SR 16 (NS) | At wk 2:  1) 11/33(33.3%) vs 4/30(13.3%) | YMRS (NS) | At 1-2 weeks:  0/33 (0%) vs  0/30 (0%) | NS | At 2 wk:  NS | NS | NS | 63/74  = 85.1% |
| Franchini et al. (2009) ^(33)^ | RCT (two-arm)  Open labeled | DSM-IV-TR | 1) HAM-D_21_ (≦8) | At wk 6:  1) 16/16 (100%) vs 10/10(100%) | Observation (NS) | At 1-6 weeks:  1/17 (5.9%) vs 0/10 (0%) | NS | NS | NS | NS | 26/27  = 96.3% |

CGI = Clinical Global Impression, DDERS = Dimensions of Delusion Rating Scale, GAF = Global Assessment of Functioning Scale, HAM-D_17_ = 17-item Hamilton Depression Rating Scale (HAM-D), HAM-D_21_ = 21-item Hamilton Depression Rating Scale (only after run-in period), Q-LES-Q = Quality of Life Enjoyment and Satisfaction Questionnaire [overall 54 items], PSQI = Pittsburgh Sleep Quality Index, QIDS-SR=16-item Quick Inventory of Depressive Symptomatology Self-Report, MADRS = Montgomery-Asberg Depression Rating Scale, mean VAS = Visual Analog Scale scores (08:00, 13:00, 18:00), SCID-I = Structured Clinical Interview for DSM-IV Axis I Disorders, YMRS = Young Mania Rating Scale, SIGH-ADS = Structured Interview Guide for the Hamilton Depression Rating Scale With Atypical Depression Supplement, SIGH-SAD = Structured Interview Guide for the Hamilton Depression Rating Scale With Seasonal Affective Disorder Supplement, # = significant difference
